# DNA-Binding Proteins Regulating pIP501 Transfer and Replication

**DOI:** 10.3389/fmolb.2016.00042

**Published:** 2016-08-11

**Authors:** Elisabeth Grohmann, Nikolaus Goessweiner-Mohr, Sabine Brantl

**Affiliations:** ^1^Division of Infectious Diseases, University Medical Center FreiburgFreiburg im Breisgau, Germany; ^2^Life Sciences and Technology, Beuth University of Applied Sciences BerlinBerlin, Germany; ^3^Center for Structural System Biology, University Medical Center Hamburg-EppendorfHamburg, Germany; ^4^Deutsches Elektronen-SynchrotronHamburg, Germany; ^5^Institute of Molecular Biotechnology, Austrian Academy of SciencesVienna, Austria; ^6^Research Institute of Molecular PathologyVienna, Austria; ^7^Lehrstuhl für Genetik, Biologisch-Pharmazeutische Fakultät, AG Bakteriengenetik, Friedrich-Schiller-Universität JenaJena, Germany

**Keywords:** conjugative plasmid, replication, copy number control, type IV secretion, broad-host-range, transfer control

## Abstract

pIP501 is a Gram-positive broad-host-range model plasmid intensively used for studying plasmid replication and conjugative transfer. It is a multiple antibiotic resistance plasmid frequently detected in clinical *Enterococcus faecalis* and *Enterococcus faecium* strains. Replication of pIP501 proceeds unidirectionally by a theta mechanism. The minimal replicon of pIP501 is composed of the *repR* gene encoding the essential rate-limiting replication initiator protein RepR and the origin of replication, *oriR*, located downstream of *repR*. RepR is similar to RepE of related streptococcal plasmid pAMβ1, which has been shown to possess RNase activity cleaving free RNA molecules in close proximity of the initiation site of DNA synthesis. Replication of pIP501 is controlled by the concerted action of a small protein, CopR, and an antisense RNA, RNAIII. CopR has a dual function: It acts as transcriptional repressor at the *repR* promoter and, in addition, prevents convergent transcription of RNAIII and *repR* mRNA (RNAII), which indirectly increases RNAIII synthesis. CopR binds asymmetrically as a dimer at two consecutive binding sites upstream of and overlapping with the *repR* promoter. RNAIII induces transcriptional attenuation within the leader region of the *repR* mRNA (RNAII). Deletion of either control component causes a 10- to 20-fold increase of plasmid copy number, while simultaneous deletions have no additional effect. Conjugative transfer of pIP501 depends on a type IV secretion system (T4SS) encoded in a single operon. Its transfer host-range is considerably broad, as it has been transferred to virtually all Gram-positive bacteria including *Streptomyces* and even the Gram-negative *Escherichia coli*. Expression of the 15 genes encoding the T4SS is tightly controlled by binding of the relaxase TraA, the transfer initiator protein, to the operon promoter overlapping with the origin of transfer (*oriT*). The T4SS operon encodes the DNA-binding proteins TraJ (VirD4-like coupling protein) and the VirB4-like ATPase, TraE. Both proteins are actively involved in conjugative DNA transport. Moreover, the operon encodes TraN, a small cytoplasmic protein, whose specific binding to a sequence upstream of the *oriT nic*-site was demonstrated. TraN seems to be an effective repressor of pIP501 transfer, as conjugative transfer rates were significantly increased in an *E. faecalis* pIP501Δ*traN* mutant.

## Introduction

Plasmids are extrachromosomal elements, by definition not encoding any essential functions for the bacterial host but rather contributing additional traits, which can be advantageous or even essential for survival under particular conditions, e.g., in the presence of antibiotic pressure. pIP501 is a considerably small, 30.6-kb broad-host-range self-transmissible plasmid, which was isolated from a clinical *Streptococcus agalactiae* strain (Evans and Macrina, 1983). It belongs to incompatibility group *Inc18* and encodes resistance to antibiotics of the macrolide/lincosamide/streptogramin (MLS) group and to chloramphenicol.

*Inc18* plasmids encode an efficient plasmid stabilization system, the ε-θ ζ locus functioning as a toxin-antitoxin system (Ceglowski et al., 1993). The ϖ–ε-ζ operon of pSM19035 and of other *Inc18* plasmids is a novel proteic plasmid addiction system in which the ε and ζ genes code for an antitoxin and a toxin, respectively, while ϖ- plays an autoregulatory role. Broad-host-range efficiency of the ϖ–ε-ζ cassette has been demonstrated in eight different Gram-positive bacteria, including among others the human pathogens, *E. faecalis, S. agalactiae, Streptococcus pyogenes*, and *Staphylococcus aureus* (Brzozowska et al., 2012). Expression of toxin Zeta was shown to be bactericidal for Gram-positive bacteria and bacteriostatic for the Gram-negative *Escherichia coli*, thus stabilizing plasmids in *E. coli* less efficiently than in Gram-positive bacteria (Zielenkiewicz and Ceglowski, 2005).

pIP501 replicons stabilized by this toxin-antitoxin system have been frequently encountered in *E. faecium* isolates from geographically diverse clinical, human community and poultry fecal origin (Rosvoll et al., 2010). In addition, pIP501-like replicons are often linked with the vancomycin resistance phenotype encoded by *vanA* (Rosvoll et al., 2010).

pIP501 is characterized by a replicon, which is tightly controlled on several levels by protein and RNA key players, and a conjugative transfer (*tra*) region comprising almost half of the plasmid genome encoding 15 putative Tra factors making up a Gram-positive T4SS. The review summarizes (i) key findings on replication and copy number control processes that involve DNA-binding proteins and (ii) current knowledge on key factors of the pIP501 T4SS whose activity involves interaction with DNA. The review ends with a Conclusion and Perspective section on urgent future research needs in the field of plasmid biology.

## pIP501 replication and copy number control

Plasmid pIP501 from *S. agalactiae* belongs, together with pAMβ1 from *E. faecalis* and pSM19035 from *S. pyogenes* to the *Inc18* family of plasmids that replicate unidirectionally by the theta mechanism (Brantl et al., 1990; Bruand et al., 1993) in a multitude of Gram-positive bacteria, including *Bacillus subtilis*. All three plasmids show a high degree of sequence identity in their replication regions (Brantl et al., 1989, 1990; Swinfield et al., 1990).

### The RepR protein

**Figure 1:**
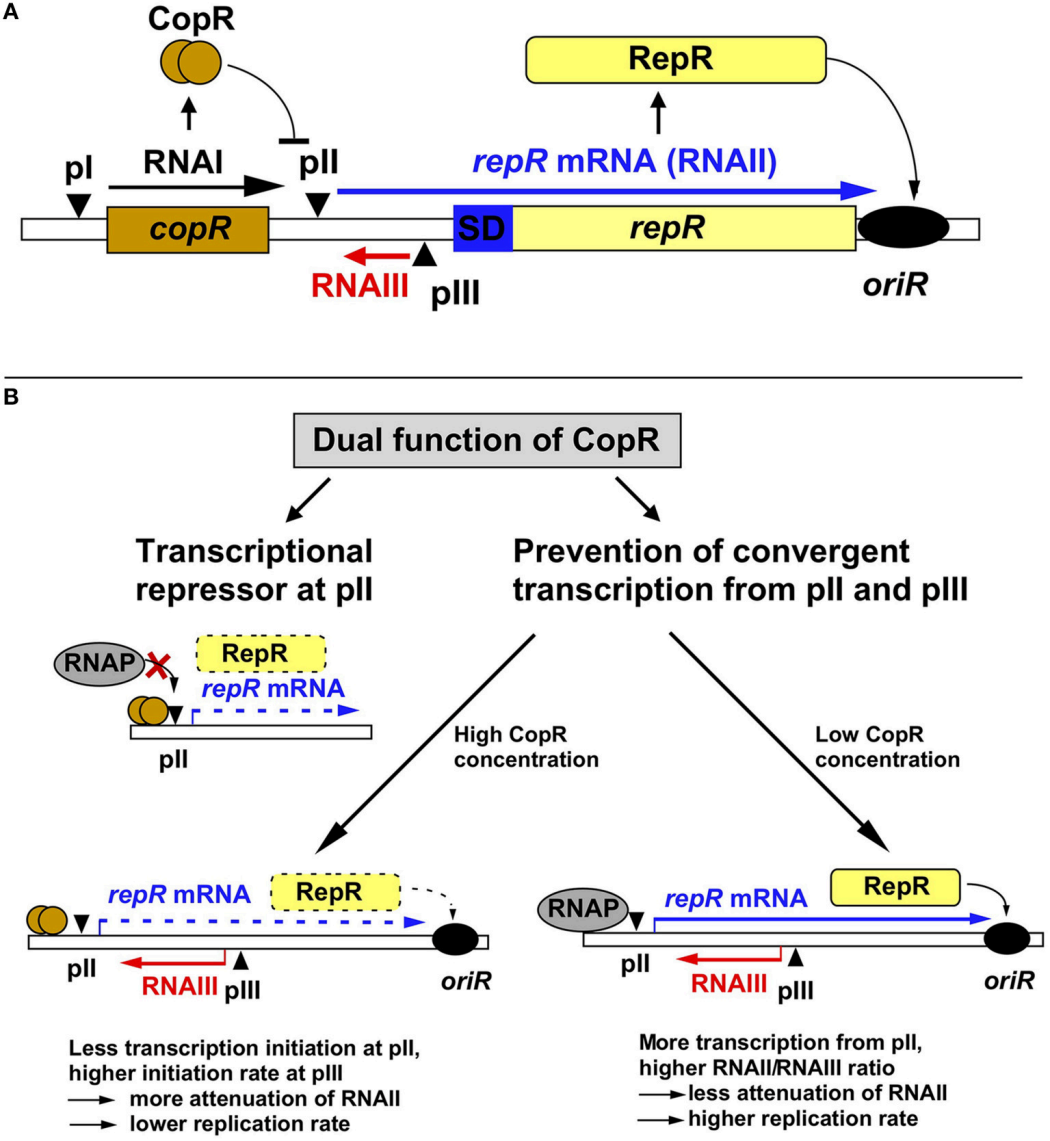
**Regulation of pIP501 replication. (A)** Working model on regulation of pIP501 replication. The minimal replicon with the *copR* and *repR* genes is shown, separated by the 329-bp long leader region. **(B)** Dual function of CopR. On the one hand, CopR represses transcription from the *repR* promoter pII by binding to its operator region upstream of the −35 box, thereby inhibiting RNAP binding. On the other hand, CopR prevents convergent transcription from pII and pIII that, in its absence reduces initiation at the supercoiling-sensitive antisense promoter pIII which results in lower RNAIII compared to RNAII levels, yielding a lower attenuation rate, and, hence, higher replication rate. Additionally, the higher amount of RNAII (*repR* mRNA) titrates the remaining long-lived RNAIII, which further reduces the amount of the inhibitor. In the presence of CopR, RNAP transcribes less frequently through pIII, which allows higher initiation rates at pIII resulting in increased premature termination of RNAII transcription and, consequently, lower replication rates. Left, the plasmid-copy number is 10-fold lower than right, but relatively more RNAIII is present, the amount of RNAIII is (as determined in Northern blots) approximately the same in both cases, reflected by the same thickness of the red arrows symbolizing RNAIII.

The minimal pIP501 replicon comprises the *repR* gene encoding the essential replication initiator protein RepR (57.4 kDa) and the replication origin, *oriR* (Brantl et al., 1990) located downstream of the *repR* gene (see Figure 1). The minimal origin *oriR* has been narrowed down to 52 bp and includes an inverted repeat, both branches of which are essential (Brantl and Behnke, 1992a). The RepR protein is rate-limiting for pIP501 replication (Brantl and Behnke, 1992c) and can both act *in cis* and *in trans* at *oriR*. The *repR* promoter pII is located 300 bp upstream of the Shine-Dalgarno (SD) sequence of the *repR* gene, and this leader region proved to be essential for replication control (see below). RepR of pIP501 has not been analyzed in detail. However, the highly similar RepE protein from pAMβ1 has been biochemically characterized (Le Chatelier et al., 2001): RepE is a monomer and binds specifically, rapidly and durably to the origin *oriE*_pAMβ1_ at a unique binding site immediately upstream of the replication initiation site. RepE binding induces only a weak bend. In addition, it also binds non-specifically to single stranded (ss) DNA with a 2- to 4-fold greater affinity than for double stranded (ds) *oriE*. RepE binding to *oriE*_pAMβ1_ causes denaturation of the AT-rich sequence downstream of its binding site yielding an open complex that is atypical: Its formation does not require multiple RepE binding sites or a strong *oriE* bending or any co-factors, and its melted region acts as substrate for RepE binding. These properties and the requirement of transcription through the origin for DNA polymerase I to initiate replication as well as a primosome to load the replisome indicate that RepE might assist primer generation at the origin. It has been hypothesized that it might cleave its own *repE* mRNA downstream of the ORF to generate the replication primer. As RepR_pIP501_ and RepE_pAMβ1_ display 97% sequence identity, it can be assumed that these characteristics also apply for RepR_pIP501_.

### Regulation of pIP501 replication by two components

Replication of pIP501 is regulated by the products of two non-essential genes, *copR* and *rnaIII* (Brantl, 2014, 2015), which control the synthesis of the rate-limiting replication initiator protein RepR (Brantl and Behnke, 1992c). RNAIII is a 136-nt long antisense RNA and CopR is a small protein composed of 92 amino acids (aa, see below). RNAIII induces premature termination (attenuation) of transcription of the essential *repR* mRNA (Brantl et al., 1993; Brantl and Wagner, 1994, 1996; Heidrich and Brantl, 2003, 2007). CopR acts as transcriptional repressor at the essential *repR* promoter pII (Brantl, 1994). Point mutations and deletions in either *rnaIII* or *copR* result in the same 10- to 20-fold increase in the copy number of pIP501 derivatives (Brantl and Behnke, 1992b). However, simultaneous deletions do not display additive effects suggesting the involvement of a limiting host factor. Surprisingly, the half-life of RNAIII is with 30 min unusually long (Brantl and Wagner, 1996). Such a long-lived antisense RNA should presumably be a poor regulator since fortuitous decreases in plasmid copy number in individual cells could only be slowly corrected, resulting in unstable maintenance. However, unstable maintenance of pIP501 derivatives was never observed, likely because the second regulator CopR in fact has a dual function and thus provides pIP501 with a strategy to cope with the risk of unstable inheritance (Figure 1). CopR exerts its effect, by the same molecular event, on two levels: transcriptional repression of *repR* mRNA synthesis (see below), and accumulation of RNAIII by prevention of convergent transcription, thereby indirectly increasing transcription initiation from the antisense promoter pIII (Brantl and Wagner, 1997). The discovery of a second function for CopR was initiated by the surprising finding that high copy number pIP501 derivatives lacking *copR* and low copy number derivatives containing *copR* produce the same intracellular amounts of RNAIII. Transcriptional pI-*lacZ* fusions revealed that CopR does not activate its own promoter pI (Brantl, 1994) and half-life measurements indicated that CopR does not affect the half-life of RNAIII. Instead, in the presence of both sense promoter pII and antisense promoter pIII *in cis*, CopR provided *in cis* or *in trans* causes an increase in the intracellular concentration of RNAIII. This effect can be attributed to the CopR protein and not the *copR* mRNA (Brantl and Wagner, 1997). Apparently, in the absence of CopR, the increased (de-repressed) RNAII transcription interferes, *in cis*, with initiation of RNAIII transcription (“convergent transcription”), yielding a lower RNAIII/plasmid ratio. The crucial factor in convergent transcription is the movement of the RNA polymerase toward or through the pIII promoter region, whereas it does not proceed through pII. Promoter pII as well as promoter pIII are supercoiling sensitive indicated by the effect of the gyrase inhibitor novobiocin on the accumulation of both RNAII and RNAIII (Brantl and Wagner, 1997). Therefore, in the absence of CopR, transcription from pII reduces initiation at pIII by inducing positive supercoils. By contrast, in the presence of CopR, promoter pII is 10-fold repressed, so that convergent transcription is mostly abolished. Consequently, more transcription from promoter pIII can be initiated resulting in increased RNAIII/plasmid ratios. Therefore, we propose the following model for pIP501 copy number control: RNAIII alone is able to adjust increases in copy number. At higher plasmid concentrations, more RNAIII is synthesized which in turn increases transcriptional attenuation of RNAII thus decreasing the replication frequency. In contrast, fortuitous copy number decreases cannot rapidly be corrected by RNAIII, since its long half-life (Brantl and Wagner, 1996) will result in high concentrations of the inhibitor, which threatens to yield a replication frequency inappropriately low for the current copy number. CopR, due to its dual function, can correct downward fluctuations of the plasmid copy number: Decreased synthesis of CopR de-represses promoter pII. This has two consequences (Brantl, 1994): (1) enhanced transcription of RNAII and (2) convergent transcription, which reduces RNAIII transcription. Both effects enhance RepR synthesis resulting in a higher replication frequency. The molecular event of pII de-repression works as an amplifier. In summary, the concerted action of two regulatory components, RNAIII and CopR, efficiently regulates pIP501 replication and ensures stable plasmid maintenance.

### Biochemical characterization of the CopR protein

#### Three *Inc18* family plasmids encode almost identical Cop proteins

Two almost identical Cop proteins with the same functions are encoded by the related streptococcal plasmids pAMβ1 (CopF) and pSM19035 (CopS) that share a high degree of sequence similarity with CopR at the aa level (Swinfield et al., 1990; Ceglowski et al., 1993): only two conservative aa exchanges at positions 51 and 80 are present (Brantl et al., 1994) and 2 additional aa (CopF) or two lacking aa (CopS) are found at the C-terminal end. To date, CopR is the best characterized Cop protein of this family. The repressor activity of CopF has been demonstrated and its operator sequence was narrowed down to a region of 31 bp (Le Chatelier et al., 1994). CopS has not been characterized in detail.

#### Identification of bases and phosphate residues contacted by CopR

**Figure 2:**
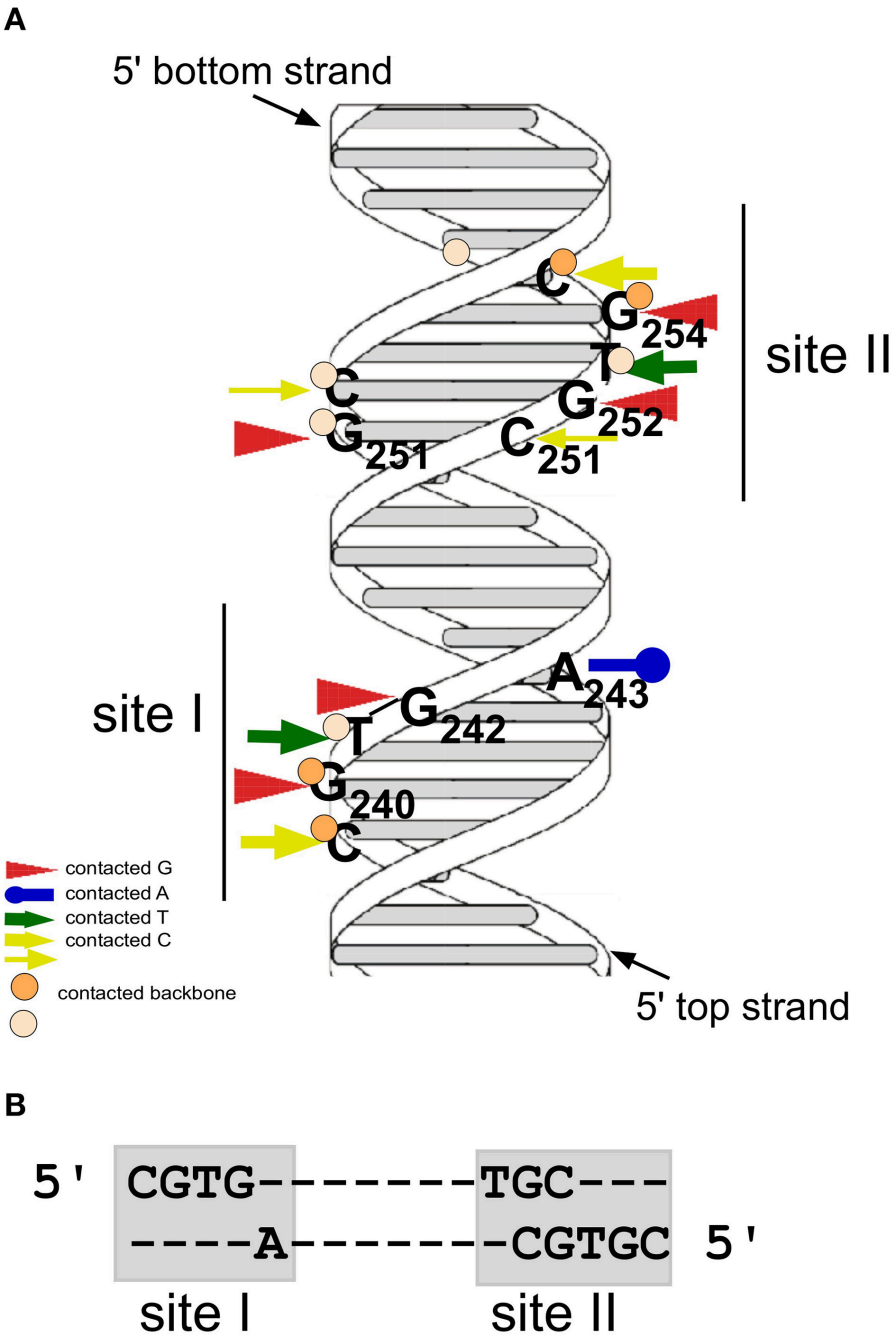
**CopR contacts two consecutive sites at the major groove of DNA. (A)** Model of the CopR DNA target with the two binding sites. Arrows denote bases contacted by CopR, circles represent phosphate groups of the DNA backbone contacted by CopR. Positions of bound Gs and As are indicated. Dark orange circles and thick yellow arrows represent strong contacts, light orange circles and thin yellow arrows weak contacts. Positions of the two binding sites are indicated. **(B)** DNA sequences of CopR binding sites I and II.

The gene product of the *copR* gene is a small protein composed of 92 aa (predicted MW 10.4 kDa) that acts as transcriptional repressor at the essential *repR* promoter pII. CopR binds to a 44-bp region containing inverted repeat IR1 upstream of pII (Brantl, 1994). It does not autoregulate its own promoter pI nor does it activate the antisense RNA promoter pIII (Brantl, 1994). To identify bases and phosphates at the backbone directly contacted by CopR, chemical footprinting studies were performed (Steinmetzer and Brantl, 1997; Figure 2A). Methylation interference identified three guanines (G240, G242, and G251) and one cytosine (C239) in the top strand and two guanines (G252 and G254) and one cytosine (C255) in the bottom strand that are contacted by CopR in the major groove of DNA (Brantl et al., 1994). Furthermore, missing base interference uncovered the contribution of the bases adjacent to these guanines to the specific DNA-protein contacts. To determine phosphate residues in the DNA backbone essential for CopR binding ethylation interference experiments were employed. In the top strand, ethylation of C239, G240, and T241 interfered strongly with CopR binding while in the bottom strand, ethylation of T253, G254, and C255 affected binding. The recognition sequence of CopR is situated at the center of inverted repeat IR1. The protein contacts two consecutive major grooves (site I and II) on the same face of the DNA. Both binding sites share the common sequence motif 5′CGTG3′, and the outermost G is most important for CopR binding. A243 and G/C251 located within the loop region of the inverted repeat IR1 evoke an imperfect symmetry within the binding sequence (Figure 2). The sequence of the CopR operator was narrowed down to 17 bp. Gel filtration and native gel electrophoresis revealed that CopR is mainly dimeric under the conditions assayed (Steinmetzer and Brantl, 1997). An initially obtained sigmoidal binding curve proved to be the result of two coupled equilibria, on the one hand dimerization of CopR monomers and on the other hand CopR dimer-DNA binding. Using analytical ultracentrifugation, a *K*_Dimer_-value of 1.44 ± 0.49 × 10^−6^ M for CopR dimers was determined (Steinmetzer et al., 1998) indicating relatively weak interactions between the two monomers. Using the *K*_Dimer−_ value and the binding curve, the equilibrium dissociation constant *K*_2_ for the CopR-DNA complex was calculated to be 4 ± 1.3 × 10^−10^ M, i.e., ≈ 0.4 nM. In this concentration range, CopR is mostly monomeric. By quantitative Western blotting, the intracellular concentration of CopR in *B. subtilis* carrying low copy number (*copR*^+^ *rnaIII*^+^) pIP501 derivatives was determined to be 20–30 μM. As this value is 10- to 20-fold higher than the *K*_Dimer_, CopR is preferentially present as a dimer in the cell. Using gel-shift assays with wild-type and a C-terminally truncated CopR species (CopΔ20), it was demonstrated that CopR also binds to the DNA as a preformed dimer (Steinmetzer et al., 1998).

#### 3D model of CopR and identification of residues involved in DNA recognition and dimerization

**Figure 3:**
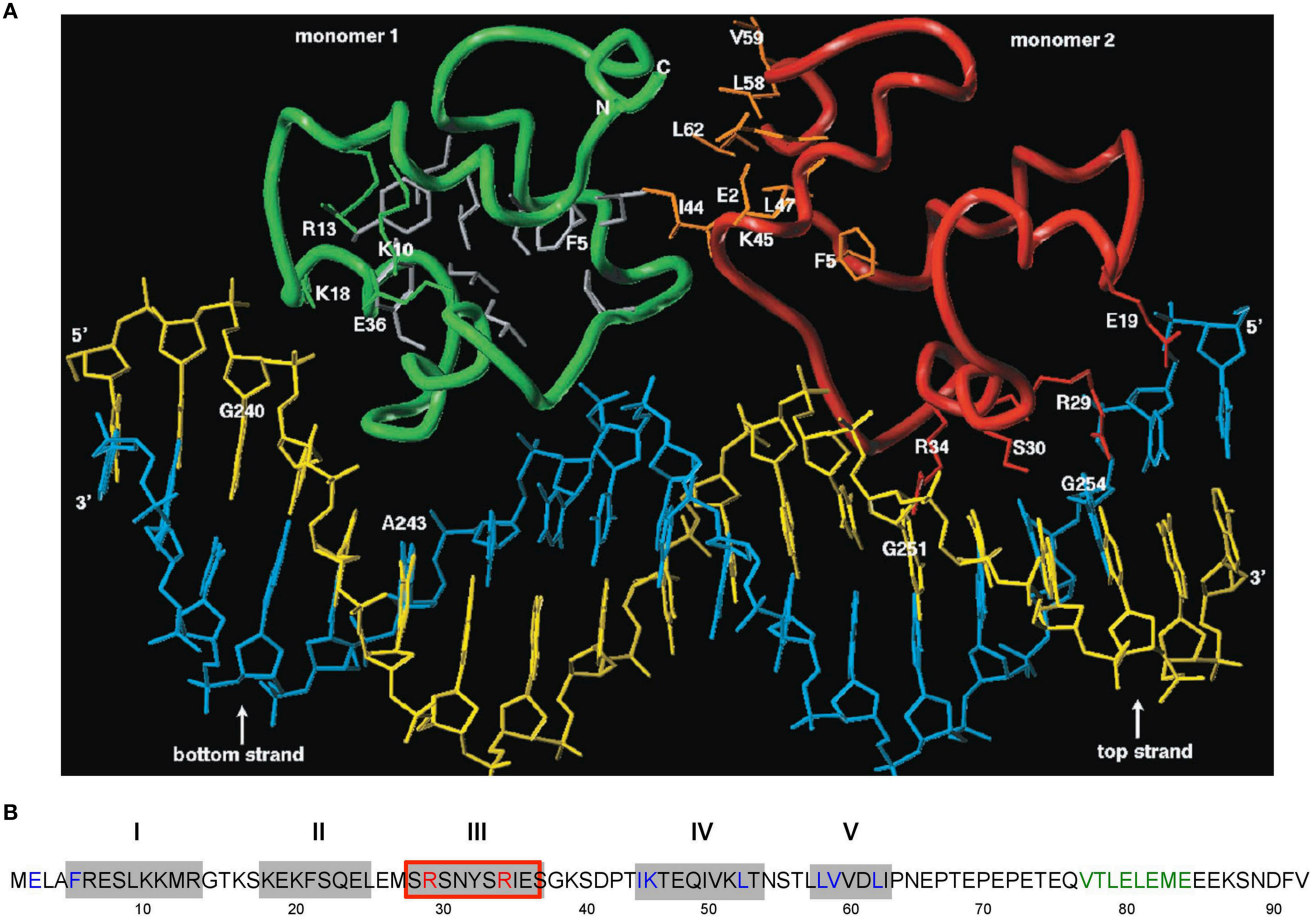
**Amino acid sequence of CopR and 3D model of the CopR-DNA complex. (A)** Model of the CopR-DNA complex. Green and red, CopR monomer I and II, respectively. Blue, DNA bottom strand. Yellow, DNA top strand. Gray, aa forming the hydrophobic core (for reasons of clarity shown only for monomer I). Orange, aa at the dimeric interface, shown only for monomer II. The presented DNA conformation is speculative. **(B)** Amino acid sequence of CopR (Brantl et al., 1994). Predicted α-helices are shown as gray boxes and numbered above the boxes. Red-framed box, DNA recognition helix. Red letters, aa involved in specific DNA recognition. Blue letters, aa predicted and shown to be involved in dimerization. Green letters, alternating hydrophobic and hydrophilic aa at the C-terminus forming a β-strand that stabilizes CopR.

A structural model encompassing the N-terminal 63 aa of CopR was constructed (Figure 3). This model was based on the rather low (14%) sequence similarity to the P22 c2 repressor (Steinmetzer et al., 2000b). The model lacks the C-terminal 29 aa that had been found previously to be not important for DNA binding. In analogy to the P22 c2 repressor this model proposes that CopR is a HTH (helix-turn-helix) protein and describes the property of the protein to bind to DNA as a dimer at two consecutive major grooves (Steinmetzer et al., 1998). The protein backbone is built up by five α-helices, two of which are involved in DNA binding. Helix I is situated between aa 5 and 13. Helix II containing aa 18–25 is proposed to be the stabilization helix, while helix III comprising aa 29–37 is suggested to be the recognition helix. Moreover, aa 44–54 and 58–62 are predicted to form helices IV and V. The model proposes that aa R29, S30, S33, and R34 in the recognition-helix contact defined bases in the DNA sequence-specifically. In addition, residues K10, K18, K20, S28, N31, and S40 are supposed to contact the DNA-phosphate backbone sequence-unspecifically. E36 is near K10, R13, and K18 that, in the model, are close together in space and in contact with the phosphate backbone of the DNA. Residues F5, L9, F21, L25, Y32, I35, P42, L47, I50, and L53 build the hydrophobic core of CopR. Residues E2, F5, I44, K45, L47, L58, V59, and L62 are located on the protein surface and are suggested to be part of the dimeric interface. The real conformation of the fifth helix involving residues L58–I63 may—due to the uncertainties in the sequence alignment—differ from the model.

Based on experimental footprinting data (Steinmetzer and Brantl, 1997), the CopR homology model and the crystal structure of the 434 c1 repressor-DNA complex, a model for the complex of CopR with the DNA target was generated (Figure 3A). To test the function of aa involved in sequence and non-sequence specific DNA recognition as well as aa important for correct protein folding, site-directed mutagenesis was employed. CD measurements of CopR variants were carried out to detect structural changes resulting from the mutations. In addition, dimerization was monitored by glutardialdehyde cross-linking and analytical ultracentrifugation. This approach allowed to localize the predicted HTH motif between aa 18 and 37 and to determine two aa within the recognition helix that make specific contacts with the DNA, R29 and R34 (Steinmetzer et al., 2000b; Figure 3A). Variants R29Q and R34Q showed only non-specific DNA binding at very high (micromolar) concentrations, while the protein structure was not affected. Furthermore, mutations of aa predicted to be involved in non-specific binding of the DNA backbone (S28T and K10Q) led to decreased binding affinity while maintaining selectivity. Additionally, substitution of aa necessary for proper folding—E36 and F5—caused significant structural changes. Taken together, these data support the model of CopR as a HTH protein that belongs to the λ repressor superfamily and uses α-helix III as recognition helix.

To verify the model predictions on aa involved in dimerization of CopR monomers, a combination of site-directed mutagenesis, EMSA, dimerization studies using sedimentation equilibrium centrifugation and CD measurements was used (Steinmetzer et al., 2000a). This allowed locating the dimeric interface between aa I44 and L62 (Figure 3A). As aa F5 situated at the N-terminus is needed for proper folding, it could not be unambigously assigned to the dimeric interface. CD measurements at protein concentrations below the *K*_Dimer_ value demonstrated that the monomer of CopR is folded. As in the first analysis, double and triple variants were constructed that all exhibited drastically increased dimerization constants, the analysis was complemented with single variants in the dimeric interface (Steinmetzer et al., 2002b). DNA binding and dimerization constants were calculated, urea-induced denaturation experiments were applied to evaluate the *in vitro* stability, and CD spectra of all mutated CopR proteins were measured. Variants I44D, L58D, V59S, and L62D had 4- to 50-fold increased *K*_Dimer_-values and bound the CopR operator only non-specifically. Thereby, the substitution of aa L58 or L62 that were predicted to form several close interface contacts severely diminished dimerization, while mutation of the weakly interacting aa V59 did not significantly affect dimerization. Whereas the CD spectra did not show drastic structural alterations, the denaturation data revealed that the four variants unfold differently compared with the wild-type. Our results reveal that the four analyzed aa are engaged in dimerization as well as in folding of the monomer, i.e., they stabilize the monomer and, in addition, the dimeric interface (Steinmetzer et al., 2002b). Possibly, for economic reasons, some aa have dual functions in a small protein like CopR. Our data obtained with the four variants carrying single amino acid exchanges indicate that conformational changes are indeed necessary for dimerization. Furthermore, we observed that a single aa can on the one hand contribute to intra-monomeric contacts, when the protein is present as a monomer, and on the other hand contribute to inter-monomeric contacts when the protein dimerizes.

#### Structure of the DNA and shape of the protein in the CopR-DNA complex

To determine the DNA conformation in the CopR-DNA complex, a combination of hydroxyl radical footprinting and fluorescence resonance energy transfer (FRET) measurements (Steinmetzer et al., 2002a) was employed. The footprints of CopR covered in total 29 bp and showed three defined areas of protection for each strand. This is comparable with the results obtained for the λ-repressor, 434-repressor, and for phage Φ105 repressor from *B. subtilis* (Tullius and Dombroski, 1986; Van Kaer et al., 1989; Ramesh and Nagaraja, 1996). The area of protection was significantly larger than that calculated earlier by chemical interference experiments, where the distance between the outermost contacts made up 17 bp (Steinmetzer and Brantl, 1997). Protected sites I and II were consistent with the previously identified contacted sites I and II. By contrast, the outer site III had not been identified before. For site III of the bottom strand, protection was weaker. This confirms our former observation that the interaction between CopR and the DNA is slightly asymmetric and also reflects the imperfect symmetry of the operator sequence (Steinmetzer and Brantl, 1997). FRET measurements revealed a bending angle of 20–25° for the DNA around the CopR protein, which is similar to that observed in the 434 c1 repressor-DNA complex and the λ c1 repressor-DNA complex. Furthermore, sedimentation velocity experiments demonstrated an extended shape of CopR dimers which accounts for the relatively large protection area detected with hydroxyl radical footprinting. To determine the global shape of the DNA in complex with CopR, FRET experiments with two DNA fragments were performed: A 19-bp DNA-fragment comprises only the minimal operator sequence (+2 bp for stabilization) and a 34-bp-fragment includes also the outer contact sites. For both fragments, bending angles of 20–25° were measured. This demonstrates that the center of the DNA bending is within the 17-bp sequence constituting the minimal operator and that the additional outer base contacts did not increase the DNA bending beyond 40–50°. Both outer binding sites do not add more than 10–15° to the overall bent. A slight bent is also in agreement with the fact that no hypersensitive sites were observed in hydroxyl radical footprinting indicating that CopR binding does not cause a drastic distortion of the DNA backbone. Analytical ultracentrifugation revealed that CopR dimers have an extended shape with a size of 8.4 nm for the fully hydrated protein. Due to this extended shape, only a gentle bending of the DNA is needed to enable CopR to make additional contacts outside of its 17-bp operator that reinforce the protein-DNA interaction. The CopR operator contains—similar to the operator of the 434 repressor—two TG-steps 11 bp apart that may constitute bending points by providing the flexibility required for the conformational changes of the DNA (Tzou and Hwang, 1999). As the CopR model does not include the 29 C-terminal aa, it can be assumed that these residues contact the outer binding sites III. Interestingly, variant CopRΔ27 lacking the 27 C-terminal aa has a 10-fold increased *K*_*D*_ value (3.8 nM instead of 0.4 nM) for the CopR-DNA complex (Kuhn et al., 2000). This corroborates the formation of additional contacts between aa of the full-length CopR-C-terminus and the DNA backbone.

#### The C-terminus of CopR is structured and important for protein stability

Previous results showed that the C-terminal 27 aa of CopR were neither necessary for DNA binding nor for dimerization (Steinmetzer et al., 1998, 2000b). However, CopRΔ27 was 5-fold impaired in copy number control *in vivo* compared to both the wild-type and CopRΔ20. Interestingly, the C-terminus of CopR is very acidic comprising 10 Glu and one Asp residues. Therefore, a series of CopR variants truncated at the C-terminus were investigated for their half-life *in vivo* as well as for dimerization, DNA binding, structure and stability *in vitro* (Kuhn et al., 2000). The last 28 aa were apparently not required for DNA binding and dimerization, although the *K*_D_ was 10-fold increased for CopRΔ27. Progressive deletions from the C-terminus significantly shortened the half-life of CopR: The half-life decreased from 42 min (wild-type CopR) over 24 min (CopRΔ7), ≈4.75 min (CopRΔ20), to ≈0.3 min (CopRΔ27). Guanidine-HCl denaturation assays corroborated that variants with shortened half-lives were also less stable *in vitro*. These results indicate that the C-terminus of CopR is required for protein stability. Amino acid substitutions within the C-terminus indicated that neither length nor charge is important for stabilization. CD measurements revealed that the C-terminus of CopR that contains alternating hydrophilic and hydrophobic aa residues is structured and forms a β-strand (Kuhn et al., 2000). Further analysis of the stabilizing motifs within the C-terminus (Kuhn et al., 2001) showed that both the wild-type (QVTLELEME, Figure 3A) and an artificial (QVTVTVTVT) β-strand structure (variant CopRVT) between aa 76 and 84 stabilized the corresponding protein derivatives. By contrast, replacement of the β-strand by an α-helix or an unstructured sequence significantly or moderately destabilized the protein. A second stabilization motif was identified in the 7 C-terminal aa, as their deletion from CopR or CopRVT reduced the half-life of the corresponding pIP501 derivatives to ≈50% (Kuhn et al., 2001). Our hypothesis is that the structured C-terminus of CopR interacts with other aa sequences in the core protein, thereby preventing its proteolytic degradation.

Surprisingly, variant CopRΔ20 with a 10-fold reduced half-life was fully functional *in vivo* in copy number control. The intracellular concentration of this variant was with 1 μM 15-fold lower than that of wild-type CopR (Kuhn et al., 2000). Why does wild-type CopR have such a long half-life, if a half-life of 4.75 min is completely sufficient for proper control? de la Hoz and colleagues investigating CopS from related plasmid pSM19035 found that the *copS* promoter is 8-fold down-regulated by the plasmid encoded ϖ- protein (de la Hoz et al., 2000). They suggested that ϖ- might represent a global regulator linking copy-number control with better than random segregation of pSM19035. As pIP501 derivatives lacking ϖ did not display defects in replication control (Brantl and Behnke, 1992b), an ϖ- homolog is apparently not required for replication control of pIP501. An 8-fold down-regulation of *copR* would still result in an intracellular CopR concentration of >2 μM, i.e., twice the amount determined for CopRΔ20. In case ϖ were included in pIP501 replication control and repressed *copR* 8-fold, the long CopR half-life would still ensure that sufficient CopR molecules are present to warrant proper control.

### Evolution of CopR resulted in maximal DNA binding affinity

When pIP501 evolved in its original host, *S. agalactiae*, selection was, apparently, for a low, but not the lowest possible, copy number, that was optimal under the conditions experienced by this bacterial host. This assumption is based on the independent *in vivo* selection of three almost identical (in their core sequences) operators of the related streptococcal plasmids pIP501, pSM19035 and pAMβ1. CopR, CopS, and CopF have similar Cop operators with identical binding sites I and II. Only the spacer regions of the *copR* and *copS* operator differ (G244A and T247A), and the flanking sequences of the *copR* and *copF* binding sites display two nt exchanges (T236G and A260G).

One instrument to adjust the copy number of pIP501 is the *K*_D_ value of the CopR-operator DNA complex. Based on the data summarized above we wondered if the *copR* operator found in nature (in pIP501) was optimized for strong DNA binding or if it would be possible to select an operator sequence that is bound more efficiently by CopR and, if yes, how such an operator would behave *in vivo*. To this end, we employed a SELEX experiment with *copR* operator sequences of different lengths combined with subsequent EMSAs with mutated operator fragments, copy-number determinations, and *in vitro* transcription (Freede and Brantl, 2004). Four experiments were performed: SELEX 1 with a randomized 7-bp spacer region, SELEX 2 with a randomized 17-bp fragment spanning the minimal operator, SELEX 3 with a longer operator (30 bp), and SELEX 4 with randomized 5-bp operator flanking regions. Our results demonstrate that the optimal spacer sequence between the two CopR binding sites comprises 7 bp, is AT rich and requires an A/T and T at the 3′ positions. By contrast, broad variations in the sequences flanking the minimal 17-bp operator did not affect CopR binding. These results show that the sequence differences between the *copR, copS*, and *copF* operator can be neglected. SELEX 2 for the minimal 17-bp *copR* operator yielded the same sequences as *in vivo* selection except that the completely symmetrical operator was found, too. Three simultaneous nucleotide exchanges outside the bases directly contacted by CopR selected in SELEX 3 did only slightly affect CopR binding *in vitro* or copy numbers *in vivo*. Therefore, we can conclude that *in vivo* evolution of the *copR* operator sequence was for maximal binding affinity.

### Transcriptional repressor CopR acts by inhibiting RNA polymerase binding

To investigate the complexes formed by the *B. subtilis* RNA polymerase (RNAP) at the *repR* promoter pII and to elucidate the mechanism exerted by CopR to repress transcription, a combination of DNase I footprinting, EMSA and KMnO_4_ footprinting was used (Licht et al., 2011). As shown by DNase I footprinting, the binding sites for CopR and RNAP overlap. EMSA confirmed that CopR and *B. subtilis* RNAP can not bind simultaneously. Instead, they compete for binding at promoter pII. Apparently, CopR prevents the access of RNAP to the promoter region by steric exclusion. We assume that CopR competes with the αCTD of the RNAP. Additionally, KMnO_4_ footprinting experiments revealed that prevention of open complex formation at pII does not further increase the repression effect of CopR. Furthermore, CopR-operator complexes were 18-fold less stable than RNAP-pII complexes in competition assays. However, due to its higher intracellular concentration CopR can effectively compete with RNAP for binding to the same region, where promoter and operator overlap. What are the consequences for copy number control? The half-lives of both CopR-pII and RNAP-pII complexes provide the time window for regulation. As CopR is produced constitutively and has a much higher intracellular concentration than the RNAP, repression can occur quickly inspite of the long half-life of the RNAP-pII complex. However, upon cell division the CopR concentration decreases, the repressor can be displaced by the RNAP—due to the much shorter half-life of the CopR-DNA complexes—and transcription of *repR* mRNA will be resumed immediately.

## pIP501 conjugative transfer

**Figure 4:**
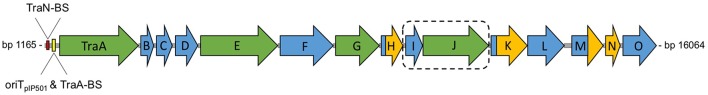
**Genetic organization of the pIP501 *tra* operon**. Genetic organization of the pIP501 *tra* operon. Proteins with known function are colored in green; the potential two-protein fusion coupling protein (consisting of TraI_pIP501_ and TraJ_pIP501_) is indicated by a dashed box. Domains or proteins which have been structurally characterized are colored in yellow; TraA binding site, TraN binding site and *oriT*_pIP501_ are indicated upstream of *traA*. The genes of the pIP501 *tra* operon are drawn to scale. BS, binding site.

pIP501 encodes a Gram-positive T4SS, whose key characteristics include the lack of a putative inner membrane transport channel owed to the different membrane composition of Gram-positive organisms and the lack of a third putative conjugative ATPase, a VirB11-like protein (Bhatty et al., 2013). The whole T4SS is encoded by the *tra* operon coding for 15 putative Tra proteins, seven of these show sequence or structural homology with Vir proteins of the Gram-negative prototype T4SS from *Agrobacterium tumefaciens* (Figure 4). Expression of the *tra* operon is controlled by the transfer initiator protein, TraA.

### Biochemical characterization of the TraA relaxase

The TraA protein belongs to the family of IncQ-type relaxases, which includes the relaxases of the Gram-positive plasmids pGO1, pSK41, and pMRC01 as well as those of plasmids RSF1010, pSC101, and pTF1 of Gram-negative bacterial origin. The prototype of this relaxase family is the MobA protein encoded by the mobilizable plasmid RSF1010. MobA is a multifunctional protein consisting of an N-terminal relaxase domain and a C-terminal DNA primase domain (Scherzinger et al., 1991; Henderson and Meyer, 1996).

To confirm the postulated relaxase activity of TraA, supercoiled plasmid pVA2241 which contains a 309-bp fragment encompassing *oriT*_pIP501_ (Wang and Macrina, 1995) was used as a substrate in an *in vitro* cleavage assay with purified TraA protein. TraA sequence-specifically cleaved the *oriT*_pIP501_ containing supercoiled DNA (Kurenbach et al., 2002). TraA relaxase activity was optimal between 42 and 45°C, with the reactions being less efficient at temperatures below 37°C. TraA-mediated cleavage of supercoiled DNA was strictly dependent on Mg^2+^ or Mn^2+^. Mg^+2^ optimum was 5 mM, and optimal Mn^2+^ concentration was 10 mM. As was the case with MobM of pMV158 (Guzman and Espinosa, 1997), the TraI-TraJ *oriT* complexes from plasmid RP4 (Pansegrau et al., 1990), TrwC from R388 (Llosa et al., 1995), and TraI of F (Matson and Morton, 1991), the maximum amount of form FII (relaxed plasmid form) produced by TraA was about 55%. Total DNA relaxation was never obtained.

Interestingly, the N-terminal part of TraA comprising the first 293 aa also cleaved supercoiled *oriT*_pIP501_ containing DNA, albeit less efficiently (Approximately 25% conversion) than the full-length protein. These data coincide with those of MobA from plasmid RSF1010. Experiments with a C-terminally truncated MobA protein demonstrated that MobA-dependent *oriT* nicking activity resides within the first 34% (243 aa) of the 78-kDa MobA protein (Scherzinger et al., 1992).

### pIP501 *tra* operon expression is not growth-phase dependent

The compact organization of the pIP501 *oriT* region is similar to that of rolling-circle-replicating plasmid pMV158, which was shown to be efficiently mobilized by pIP501 (van der Lelie et al., 1990; Kurenbach et al., 2003). The two regions are similar, meaning that the *oriT nic*-region, where the relaxase binds to its cognate DNA (Grohmann et al., 1999), lies within the respective promoter region (Farías et al., 1999). This configuration suggests autoregulation of the putative pIP501 *tra* operon consisting of the genes *traA* to *traO* (Figure 4) by the DNA relaxase TraA.

To study co-transcription of *traA* to *traO*, we conducted Reverse Transcription PCR (RT-PCR) with RNA isolated from *E. faecalis* (pIP501) cells harvested during mid exponential growth phase. Primer pairs were selected to amplify two successive genes of the *tra* region. RT-PCR resulted in products of the expected size (Kurenbach et al., 2002, 2006). We also tested for the existence of transcription products beyond *traO* using primers which would generate a *traO*/*copR* product of 480 bp. Using RNA as template, the respective product was never observed. Transcription of the pIP501 *tra* operon appears to be terminated by a strong rho-independent transcriptional terminator (Kurenbach et al., 2006).

To test the potential impact of the growth phase on the transcription of the *tra* genes, total RNA from *E. faecalis* (pIP501) was isolated at three different time-points: in the early exponential, the mid exponential, and the stationary growth phase (*OD*_600_ = 1.0). First, we looked if the *tra* genes are transcribed in all three growth phases. The selected RT-PCR amplicons from *traC* to *traD, traF* to *traG*, and *traM* to *traN* were generated with RNA from all three time-points (Kurenbach et al., 2006). Semi-quantitative RT-PCRs were carried out for *traA* to *traB*, and for *traM* to *traN* to study the transcription levels of different *tra* genes under differing physiological conditions. As a control, the constitutively expressed GAP-DH gene was amplified by RT-PCR, with RNA from *E. faecalis* cells harvested at the respective time-points as template. Densitometric analysis of the amplification products did not show any significant differences with respect to the growth phase, the same picture was obtained, as expected, for the constitutively expressed GAP-DH (Kurenbach et al., 2006).

However, we cannot exclude that *tra* gene transcription declines at a later stage in stationary phase, as we have seen slightly lower transfer frequencies (2- to 3-fold decrease) for donors and/or recipients at high cell densities (*OD*_600_ > 1) (Kurenbach et al., 2006). However, a phenomenon like “F2 phenocopies,” meaning that F^+^ cells get transfer-deficient in stationary phase (Hayes, 1964), was not observed. Transcription of several F-encoded *tra* genes decreases in mid-exponential or stationary phase, which is in agreement with a rapid decrease in transfer frequency in mid-exponential phase (Frost and Manchak, 1998). We conclude that the pIP501 *tra* genes are transcribed during the whole growth cycle of *E. faecalis* and that their level of expression does not depend on the growth phase.

### TraA relaxase binds to the P*_*tra*_* promoter

**Figure 5:**
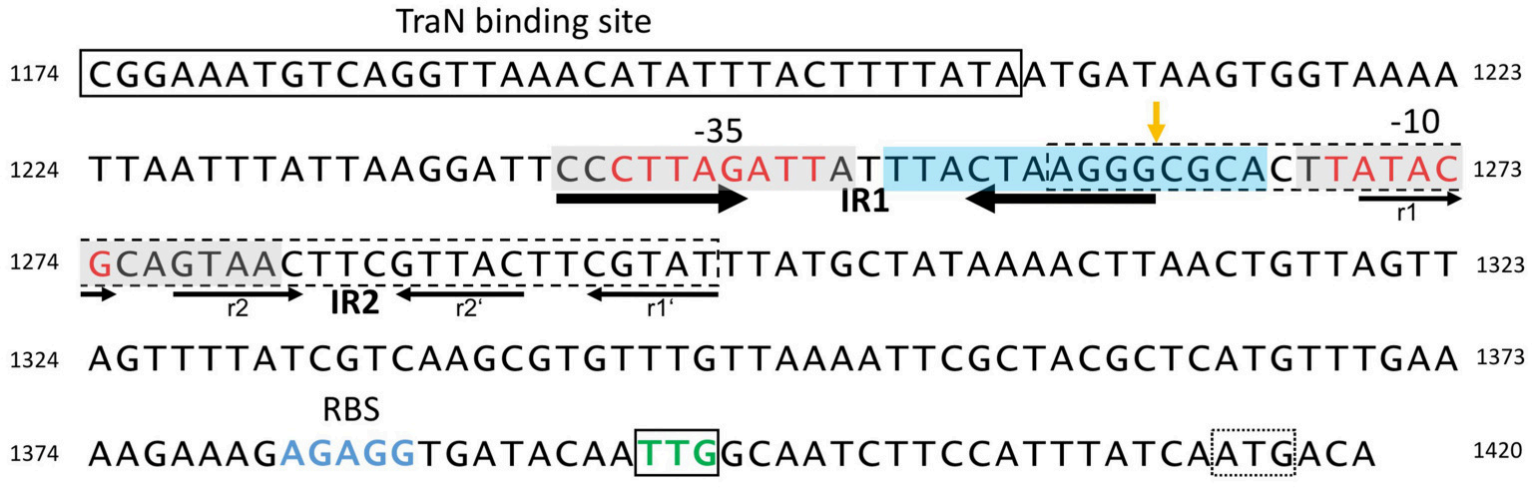
**Structure of the pIP501 *oriT* region on DNA level**. *oriT* region of pIP501 (nucleotides 1174–1420, GenBank L39769.1). Putative −10 and −35 promoter elements are colored in red; the TraN binding site is boxed. The perfect IR (IR1) is indicated with thick arrows; the second, imperfect IR (IR2) is marked with thin arrows—its repeating segments are individually named. Both repeats have the potential to generate a secondary structure. The areas that showed to be either protected from (gray box, mainly on the cleaved strand) or hypersensitive to (blue box, mainly on the non-cleaved strand) DNaseI cleavage in TraA footprinting assays (Kurenbach et al., 2006) are marked. *oriT*_pIP501_ core sequence (GenBank L39769.1, bp 1259–1296) is indicated with a dashed box; *oriT nic*-site is shown by an orange vertical arrow. *tra* operon ribosomal binding site (RBS) is colored in blue; the start codon of the first *tra* gene (*traA*, TTG) is boxed and colored in green; a second, potential start codon (ATG) is marked with a dotted box.

The compact structure of the pIP501 *oriT* region (Figure 5), in the sense that the P_*tra*_ −10 and −35 boxes overlap with the left half repeat of inverted repeat structures (IR-1 and IR-2), likely representing the TraA recognition and binding site (Kopec et al., 2005), suggests autoregulation of the *tra* operon by TraA relaxase. To study TraA binding to the P_*tra*_ promoter, three DNA fragments were selected, the first comprising the −35 and −10 region, the second only the −35 region, and the third the −10 region alone. The shortest N-terminal TraA portion exhibiting relaxase activity, TraAN_246_ (Kopec et al., 2005), was used in band shift assays with ds oligonucleotides comprising the different parts of the P_*tra*_ promoter. Applying increasing TraAN_246_ concentrations to the −10 fragment, we detected one retarded DNA–protein complex. Binding affinity for the −35 region and for the whole promoter region was weaker than for the −10 region fragment. This could be due to presence of the complete left half repeat of IR-2 in the −10 region fragment. This complete left half repeat was present in all ss oligonucleotides that bound TraAN_246_ and TraA. An oligonucleotide similar to the −10 region fragment, but additionally comprising the right half repeat, resulted in similar binding affinity (Kopec et al., 2005). For all tested promoter fragments, TraA exhibited similar binding affinities as its N-terminal domain TraAN_246_. We conclude that TraA relaxase binds to the P_*tra*_ promoter region and that only the N-terminal TraA relaxase portion, TraAN_246_, is required for efficient binding (Kopec et al., 2005).

DNase I footprinting with a 250-bp DNA fragment encompassing P_*tra*_ and the complete IR-1 and IR-2 sequences demonstrated protection of the −35 and −10 region, with hypersensitive sites on the non-cleaved strand in vicinity of the *nic* site, at the *nic* site and two nucleotides 3′ of the −10 region (Kurenbach et al., 2006). DNase I protection on the cleaved strand extended eight nucleotides to the *nic* site, the *nic* site itself appeared as hypersensitive site. The DNase I hypersensitive sites are likely due to a conformational change of the *oriT* region induced by TraA binding, making the DNA better accessible for DNase I attack.

We have demonstrated that the left half repeats of IR-1 and IR-2 are the preferential binding sites for TraA. We postulate that binding of TraA to its target DNA is required for recognition and cleavage of DNA at the 5′-GpC-3′ dinucleotide in the *nic* site, which would remain accessible to the enzymatic activity of TraA.

### Expression of the *tra* genes is controlled by TraA relaxase

To confirm that TraA binding to the P_*tra*_ promoter region affects promoter activity, we cloned the promoterless *lacZ* gene in plasmid pQF120 under control of P_*tra*_. *E. coli* cells with the construct, pQF120-P*tra*::*lacZ*, gave blue colonies on LB X-Gal plates and generated β-galactosidase activity of 401 Miller units (Kurenbach et al., 2006). The impact of *traA* expression *in trans* on P_*tra*_ activity was studied by co-transformation of *E. coli* with pQF120-P*tra*::*lacZ* and pACYC184-P*tac*::*GST-traA* which expresses *traA* under control of the *tac* promoter. Upon induction of *traA* expression β-galactosidase activity dropped to 6 Miller units. As a control, the effect of co-resident pACYC184-P*tac*::*GST* on P_*tra*_ activity of pQF120-P*tra*::*lacZ* was analyzed. No significant change in β-galactosidase activity (407 Miller units) was observed. The data clearly demonstrated that the *tra* operon is regulated at the transcriptional level by TraA relaxase (Kurenbach et al., 2006).

For Mob, the mobilization protein encoded by the mobilizable broad-host-range plasmid pBBR1, whose *nic* site is identical with that of pMV158, autoregulation by binding of Mob to its promoter region overlapping with *oriT* has also been demonstrated (Szpirer et al., 2001).

Autoregulation of *tra* gene expression mediated by the transfer initiator protein, the DNA relaxase, seems to be an efficient mechanism to shut down plasmid transfer at a very early stage of conjugation, and is likely destined to obtain an optimum balance between the maximum transfer potential and the lowest burden for the host.

### Two conjugative ATPases show non-specific DNA binding activity

The pIP501 *tra* region comprises a Gram-positive conjugative T4SS, encoding, like most of the related systems in Gram-positive bacteria, two ATPases, VirB4-like ATPase, TraE, and VirD4-like coupling protein, TraJ (for a recent review on pIP501 T4SS see Goessweiner-Mohr et al., 2013, 2014a). However, in contrast to coupling proteins from other T4SSs from Gram-negative and Gram-positive bacteria alike, pIP501 appears to encode the first coupling protein consisting of two proteins, the TraJ protein and the TraI protein. TraJ has ATP-binding and low ATPase activity *in vitro*, and the membrane-associated TraI protein, encoded immediately upstream of *traJ* in the *tra* operon (Figure 4), presumably recruits TraJ via protein-protein interaction to its putative site of action, the cytoplasmic membrane.

Already in 2002, Llosa and coworkers postulated that during conjugative plasmid transfer, ss-plasmid DNA is “actively pushed into the recipient cell by action of the coupling protein” (Llosa et al., 2002). In the case of pIP501, the energy for this process could be generated by ATP hydrolysis, mediated by TraJ. EMSAs with purified TraJ on ssDNA and dsDNA containing the minimal *oriT*_pIP501_ region (GenBank L39769.1, bp 1259–1296) or random DNA of the same size with no similarity to *oriT*_pIP501_ were performed. The DNA substrates were 42 bases or 42 bp long; the random 42-mer lacked the ability to form a hairpin-like secondary structure (Kopec et al., 2005), one of the prototypical characteristics of *oriT* regions. TraJ bound non-sequence specifically to both ssDNA substrates, whereas binding to dsDNA substrates was not observed, not even at very high TraJ concentrations (Arends, 2010). These observations are in agreement with the postulated function of TraJ as a conjugative coupling protein, connecting the relaxosome consisting of the TraA relaxase covalently bound to the 5′-end of the processed ss pIP501 DNA with the mating pair formation complex.

The VirB4-like ATPase, TraE, which showed higher *in vitro* ATPase activity than the coupling protein (Çelik, 2011) also bound non-sequence specifically to ss *oriT* 42-mer DNA and random 42-mer DNA in EMSAs performed similarly to those described above for TraJ. As demonstrated for TraJ, dsDNA was no substrate for TraE (Çelik, 2011). TraE and TraJ could be both actively involved in generating energy for the T4SS process, presumably each ATPase producing energy for different step(s) in the T4S process. Details on these processes have not been unraveled so far.

### TraN is a putative transfer repressor

#### TraN binds sequence-specifically to *oriT*_pIP501_ DNA

TraN is a small (14.4 kDa) soluble cytoplasmic protein encoded by *traN*, the penultimate gene of the pIP501 *tra* operon. The structure of TraN was solved to 1.35 Å resolution. It contains an internal dimer fold with antiparallel β-sheets in the center and a HTH motif at both ends (Goessweiner-Mohr et al., 2012, 2014b).

Because TraN co-purified with DNA, we investigated if it can interact with radiolabelled ssDNA and dsDNA oligonucleotides. By applying the identical oligonucleotides as described for the EMSAs with TraE and TraJ, TraN showed only a slight shift for the ssDNA oligonucleotides, whereas the dsDNA fragments were significantly shifted. The random and the *oriT*_pIP501_ containing oligonucleotide showed the same binding affinity (Goessweiner-Mohr et al., 2014b).

To search for a potential sequence-specific TraN binding site, we conducted EMSAs with dsDNA fragments encompassing the *oriT*_pIP501_ and sequences upstream and downstream of this region. At high TraN concentrations, all DNA fragments were cooperatively shifted. A small but significant stepwise shift using an equimolar protein:DNA ratio was visible only for fragments comprising a common 149-bp sequence 5′ of the *oriT* sequence, for which we postulate a preferred TraN binding site (Goessweiner-Mohr et al., 2014b).

To delimit the specific TraN binding site within the 149-bp sequence, we designed a new footprinting assay which is based upon 5′-to-3′ exonuclease digestion. The TraN binding site was localized to a 34-bp sequence located 55 bp 5′ of *oriT*_pIP501_ *nic*-site. Interestingly, the TraN binding site has no direct or inverted repeats but is A/T rich (Goessweiner-Mohr et al., 2014b).

Thermal stability of TraN was studied in presence and absence of DNA with a Thermofluor-based assay. The melting temperature (Tm) of TraN alone amounted to 54.3°C; the binding of a non-specific (random) 34-mer dsDNA oligonucleotide raised the Tm to 65.2°C, whereas addition of DNA containing the specific binding site increased Tm to 70.4°C. The stabilizing effect indicates an enhanced binding affinity for the specific site compared with the random DNA (Goessweiner-Mohr et al., 2014b).

To determine whether there is a difference in the molar ratio of the TraN–DNA interaction between the random and the specific oligonucleotides, as well as to obtain information on the respective binding constants, isothermal titration calorimetry analyses with the oligonucleotide encompassing the binding site and the non-specific (random) oligonucleotides used in the Thermofluor experiments were carried out. When titrating with non-specific DNA, two TraN molecules bound to one dsDNA fragment (in a 2:1 ratio), whereas, as expected, equimolar stoichiometry (1:1 ratio) was observed for the specific interaction. TraN was found to bind to the specific binding site exothermically with a binding constant of 10^7^ M^−1^ in comparison to endothermic binding to the non-specific sequence with a binding constant of 10^5^ M^−2^ (2:1 binding ratio; Goessweiner-Mohr et al., 2014b).

#### The crystal structure of the TraN-DNA complex has been solved to high resolution

Recently, we solved the 1.9 Å co-crystal structure of TraN bound to its specific 34-bp binding site upstream of the *oriT*_pIP501_*nic*-site, described above (Goessweiner-Mohr et al., in preparation). The binding mode postulated in Goessweiner-Mohr et al. (2014b) could be confirmed: “The recognition helices of the two mirrored HTH motifs enter two adjacent major grooves of the dsDNA binding site.” Furthermore, the tip of the loops between strands 2 and 3 as well as strands 5 and 6, which are close to the internal 2-fold axis, are interacting with the minor groove. While tied to its binding site, TraN slightly bends the dsDNA oligonucleotide used in the crystallization setup (Goessweiner-Mohr et al., in preparation).

#### TraN is not an essential T4SS protein

Very recently we generated a markerless *E. faecalis* JH2-2 (pIP501Δ*traN*) mutant by applying a two-step recombination technique developed for construction of mutants in *E. faecali*s (Kristich et al., 2007). Surprisingly, in standard *in vitro* mating tests we could demonstrate that TraN is not an essential T4SS protein but contrary to expectations, *traN* deletion resulted in an increase of pIP501 transfer efficiency.

#### TraN shows structural homology with transcriptional regulators: potential role of TraN in the T4S process

In searches for proteins structurally similar to TraN we only found hits that resemble one half of the protein. Amongst others, the TraN fold resembles that of the N-terminal domain of transcriptional regulators of the MerR family (Goessweiner-Mohr et al., 2014b), for example a transcriptional activator from *Bacillus thuringiensi*s (PDB entry 3gpv; New York SGX Research Center for Structural Genomics). Transcriptional activators of the MerR family comprise an N-terminal winged-helix DNA-binding domain and recognize the specific DNA site as a dimer where the recognition helices of the HTH motifs are inserted into two adjacent major grooves.

The dimerization motif of MerR proteins is completely distinct from the internal dimer configuration of TraN, which requires hydrophobic interactions within a barrel-like motif in its center. Contrarily to MerR family proteins, which contain a C-terminal effector-binding region (Brown et al., 2003), neither in TraN nor TraN-like proteins of related T4SSs such a C-terminal extension was found. All TraN-like proteins found are of enterococcal origin (from conjugative *E. faecalis* plasmids, pRE25 and pAMβ1, *E. faecium* plasmid pVEF3 and two genomically located TraN-like proteins from an *E. faecalis* and *Enterococcus italicus* strain), and their sequence is highly similar to that of TraN. All other proteins found (transposon or bacteriophage-encoded excisionases and MerR family proteins) have only a single TraN-like domain (Goessweiner-Mohr et al., 2014b).

Due to the structural similarity of TraN with MerR-like transcriptional regulators and the fact that *traN* deletion resulted in a 2 log increase of pIP501 transfer efficiency, we postulate that TraN could repress pIP501 transfer by regulating either expression of the pIP501 *tr*a operon or TraA activity.

Although MerR-like proteins show only similarity to the fold of a single TraN domain, binding to DNA requires the formation of a homodimer (PDB entry 3gpv) which binds to two adjacent major grooves of dsDNA, as postulated for TraN. Expression of the pIP501 *tra* genes is already autoregulated by TraA relaxase (Kurenbach et al., 2006), TraA binds to the two left half repeats of IR1-1 and IR1-2 (Kurenbach et al., 2006) which overlap with the −10 and −35 box of the P_*tra*_ promoter respectively. Specific DNA recognition and binding is required for TraA-mediated site-specific nicking at the 5′-GpC-3′ dinucleotide in the *nic*-site (nucleotide position 1262/1263, Acc. Nr. L39769) which thus will be accessible to the enzymatic activity of TraA (Kurenbach et al., 2006; see also Figure 5). TraN could act as an additional repressor of the *tra* operon by specifically binding to a 34-bp sequence located 55 bp upstream of the *nic*-site thereby inhibiting RNA polymerase from efficient transcription of the *tra* operon. We hypothesize that this negative regulation could be relieved by binding of putative interaction partners, e.g., TraE or TraJ (Abajy et al., 2007) to TraN, possibly as response to (i) the presence of potential recipient cells/mating partners or (ii) an assembled putative pIP501 T4SS core complex. Experimental studies on the mechanism of TraN regulation are in progress.

## Speculations on control of pIP501 transfer gene expression

Tight control of *tra* gene expression is a general feature of mobile genetic elements from Gram-negative and Gram-positive bacteria alike, presumably to ensure that costly—referring to bacterial fitness—expression of multiple Tra proteins only takes place when the effort is worthwhile because potential recipients are present or more generally speaking the environmental conditions allow efficient plasmid transfer. Different modes of controlling conjugative transfer are known: The well-characterized Gram-negative conjugative broad-host-range *IncP* plasmids, F plasmid and F-related plasmids, such as R1 and R100, have a very complex regulation system controlling expression of Tra factors at transcriptional and translational level involving not only plasmid-encoded factors but also host-factors (Zatyka et al., 1994, 1997; Taki et al., 1998; Adamczyk and Jagura-Burdzy, 2003; Will and Frost, 2006; Wong et al., 2012). Additionally, in case of TraJ from plasmid R1 and F-related plasmids, regulation of the transfer operon via a sense/antisense RNA system has been shown (Koraimann et al., 1991, 1996; Mark Glover et al., 2015). For Gram-positive bacteria, the sex-pheromone-responsive enterococcal plasmids, particularly pCF10, are those with the best studied regulatory processes controlling conjugative transfer (Tanimoto et al., 1996; Muscholl-Silberhorn, 2000; Dunny, 2007, 2013; Folli et al., 2008).

None of the known conjugation control systems fits to what we have observed for broad-host-range plasmid pIP501. pIP501 *tra* gene expression seems to be always on, independent of the growth phase of the host (Kurenbach et al., 2006) and presence of potential recipients, presumably at a low basic level. *tra* gene expression was shown to be controlled by the transfer initiator protein, TraA, which regulates its own synthesis and that of the other Tra factors by binding to the P_*tra*_ promoter overlapping with *oriT* (Kurenbach et al., 2006; see also Figure 5).

**Figure 6:**
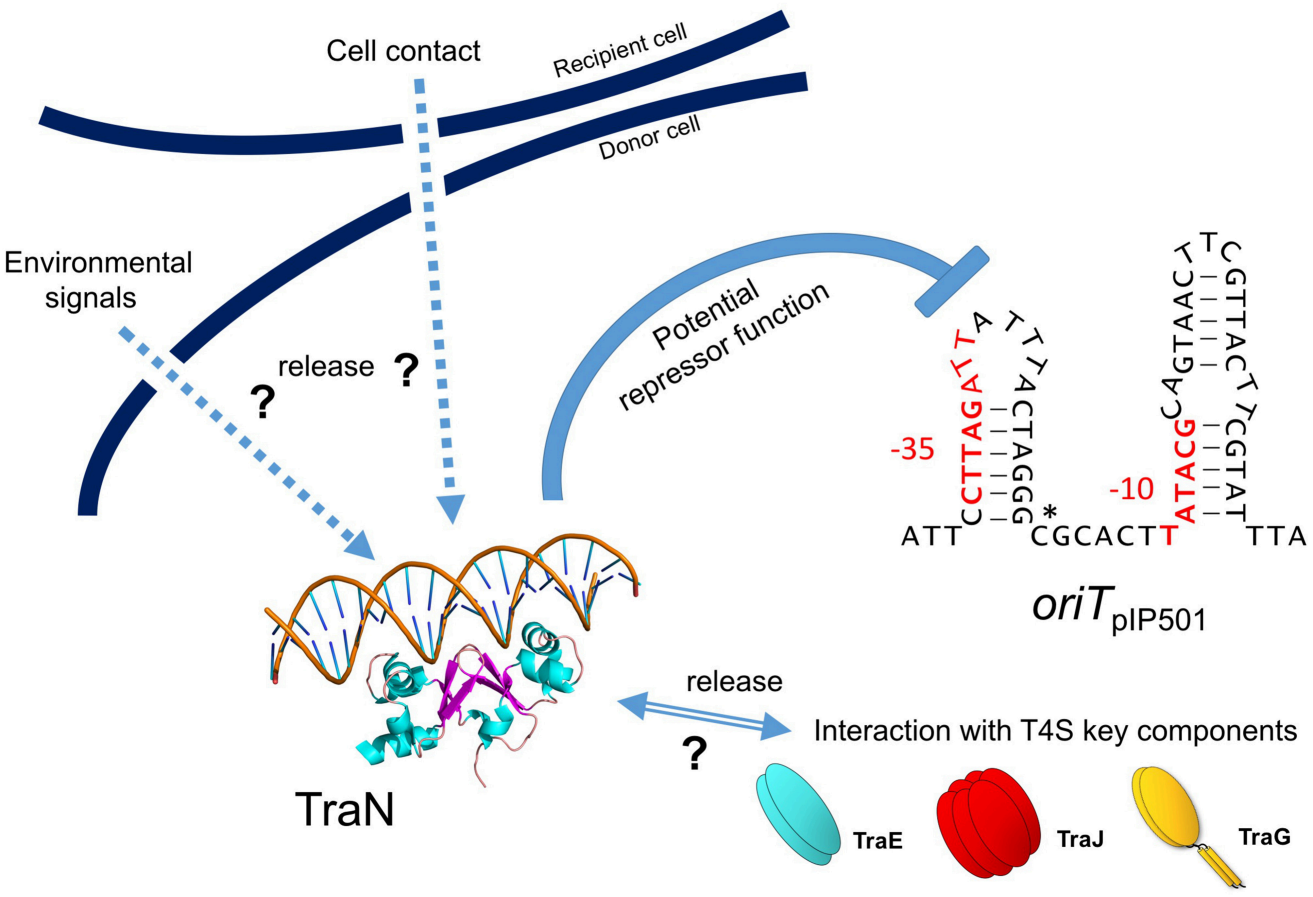
**Model for negative *tra* operon regulation by potential transcriptional repressor TraN**. TraN is shown docked to a DNA strand, with two helices reaching into two adjacent major grooves and two loops in the center of TraN extending into the minor groove in between the major grooves (adapted from Goessweiner-Mohr et al., 2014b; Figure S6). *oriT*_pIP501_ is depicted as a stem-loop structure as potentially generated by the two IR sequences (see also Figure 5). Putative −10 and −35 *tra* promoter regions are colored in red; *oriT nic*-site is marked by a star. Potential TraN repressor function is indicated; hypothetical release signals are denoted by arrows (environmental signals, recipient cell contact, interaction with T4S key components—TraE, TraJ, TraG).

Recently, we detected binding of another Tra factor, TraN, to a region 55 bp upstream of the *oriT nic*-site (Goessweiner-Mohr et al., 2014b; Figure 5 in this article). This TraN-binding site is located only 35 bp upstream of the −35 region of the P_*tra*_ promoter. Thus, we postulate that the *tra* operon might be negatively controlled by two proteins, the TraA relaxase binding to the −10 and −35 region of the promoter thereby leaving the *oriT nic*-site accessible for specific TraA cleavage and by the winged-helix-turn-helix DNA-binding protein TraN, binding to a unique operator site (present only once on the pIP501 genome) upstream of P_*tra*_ promoter. We postulate that TraA activity is blocked by binding of TraN upstream of the *oriT nic*-site. Either by (i) receiving environmentals signals which could include the presence or already the contact of the donor cell with a potential recipient cell and/or (ii) by interaction of TraN with T4S key components, such as TraE, TraG or TraJ (binding to these proteins has been observed in the yeast two-hybrid system Abajy et al., 2007), TraN would be released from the DNA, likely resulting in a conformational change of the DNA in the vicinity of the TraA binding site triggering *nic*-cleavage by TraA. Our working model of pIP501 *tra* operon regulation is depicted in Figure 6.

A putative winged-helix-turn-helix DNA-binding protein, RctA from the symbiotic rhizobial megaplasmids, has been demonstrated to repress transcription of conjugative transfer genes of pRetCFN42d, the symbiotic plasmid (pSym) of *Rhizobium etli* (Pérez-Mendoza et al., 2005; Sepúlveda et al., 2008; Nogales et al., 2013).

The negative regulation of pIP501 *tra* gene expression exerted by two (putative) transcriptional regulators would be in agreement with the generally low transfer frequencies of pIP501, in the range of (2 − 8) × 10^−5^ transconjugants per recipient for intraspecies *E. faecalis* matings (Arends et al., 2013; Fercher et al., 2016).

## Conclusions and perspectives

Conjugative transfer of diverse genetic traits, such as antibiotic or heavy metal resistance genes, virulence genes or genes conferring specific metabolic capabilities such as degradation of anthropogenic compounds is a natural process going on everywhere in nature at diverse transfer rates, as these naturally depend on the plasmids involved and on the habitat. Availability of nutrients and water, or in other words, good physiological conditions of donor and recipients, are generally accepted as conditions favoring horizontal plasmid transfer. Availability of colonizable surfaces for the microorganisms is another very important feature, as the close proximity of microorganisms in surface-associated communities, the so-called biofilms, increases the chances of horizontal gene exchange (Hausner and Wuertz, 1999; Sørensen et al., 2005; Madsen et al., 2012).

The observation that *tra* gene expression seems to be a tightly controlled process not only holds true for plasmids of the *Inc18* group, but seems to be a general feature of self-transmissible plasmids of diverse origin. In particular, the expression of relaxosome components seems to be tightly regulated, in many plasmids it is under autoregulatory control by the relaxase or relaxase accessory proteins. One of the most complex regulatory circuits controlling the production of relaxosome proteins has been deciphered in the prototype Gram-negative broad-host-range plasmid RK2. Zatyka and coworkers argued that the complex regulatory circuits involved in regulation of IncPα plasmid RK2 provide an autoregulatory way of ensuring production of enough relaxosome proteins without overburdening the host (Zatyka et al., 1994). Expression of the *tra* genes of F-plasmid is also tightly controlled by a number of factors, including among others, a plasmid-encoded activator and two autoregulators. One of them, TraM, is a component of the F relaxosome (Will and Frost, 2006). In all these plasmids the level and stringency of the regulatory processes appear to be in good balance with the transfer potential of the host in order to reduce its fitness costs.

Detailed knowledge of these regulatory processes in Gram-positive bacteria is still scarce, thus challenging tasks of the coming years will be to unravel the internal as well as external environmental signals triggering plasmid transfer on the molecular level to develop more efficient interference strategies to efficiently reduce conjugative spread of antibiotic resistance genes.

With regard to replication of pIP501, the identification of a third (upper) regulatory level would be required to unravel why deletion of both regulatory components, CopR and RNAIII, does not show an additive effect. In the related plasmid pSM19035, ϖ protein was found to be this central regulator (de la Hoz et al., 2000). However, in pIP501, no promoter has been detected so far preceding *orf* ϖ.

Thus, for both, pIP501 replication and transfer, the most urgent questions to answer concern global regulatory processes governing the success of pIP501-like multiple antibiotic resistance replicons in terms of maintenance and wide spread, particularly, in hospital environments.

## Author contributions

EG, NG, and SB contributed to writing of the manuscript. NG, SB designed the figures, all authors approved the final version of the manuscript.

### Conflict of interest statement

The authors declare that the research was conducted in the absence of any commercial or financial relationships that could be construed as a potential conflict of interest. The reviewer GS and handling Editor declared their shared affiliation, and the handling Editor states that the process nevertheless met the standards of a fair and objective review.
